# Exploring Patient, Proxy, and Clinician Perspectives on the Value and Impact of an Inpatient Portal: A Reflexive Thematic Analysis

**DOI:** 10.2196/52703

**Published:** 2024-11-20

**Authors:** Simone Schmidt, Adam Boulton, Benita Butler, Timothy Fazio

**Affiliations:** 1 EMR Team Royal Melbourne Hospital Melbourne Australia; 2 School of Computing and Information Systems University of Melbourne Melbourne Australia; 3 Department of Medicine, Melbourne Medical School University of Melbourne Melbourne Australia

**Keywords:** inpatient portal, patient perspective, clinician perspective, information sharing, clinician-patient relationship, person-centered care, reflexive thematic analysis, qualitative research, mobile phone

## Abstract

**Background:**

Research exploring perspectives on inpatient portals reports that patients desire the information affordances of inpatient portals, and clinicians recognize their value for improving patient experience but also express caution regarding sharing aspects of the medical record. This study contributed to the existing literature on inpatient portals by considering the psychosocial dimension of clinician resistance to information sharing with inpatients and the power dynamic associated with clinician-patient information asymmetry. Along with the information affordances commonly discussed in this area, this study explored perspectives on the novel option to audio record consultations via an inpatient portal.

**Objective:**

This study aims to understand patient, proxy, and clinician perspectives on the value and impact of an inpatient portal within the Australian context. It explores clinician resistance and receptivity to sharing aspects of the medical record with patients and the power dynamic that characterizes the relationship between clinician and patient. It considers how an inpatient portal might assist in the transformation of this relationship such that this relationship could be characterized by greater information symmetry.

**Methods:**

Interviews were conducted with patients (n=20), proxies (n=4), and clinicians (n=21) recruited from 3 areas within the Royal Melbourne Hospital, where the portal would later be implemented. A largely inductive reflexive thematic analysis was conducted.

**Results:**

Patient and proxy participants reported that they wanted to understand what is happening in their care for peace of mind and that an inpatient portal could support this understanding. Clinician participants reflected on how they might transform their information-sharing practice to provide greater transparency in their relationship with patients. Participants considered the types of information that could be shared and how this information could be shared via an inpatient portal. Four key themes were generated: (1) affording the patient and proxy awareness, control, and reassurance through sharing accessible and meaningful information; (2) protecting the clinician and safeguarding quality health care in information sharing; (3) flexibly deploying the functions depending upon clinician, patient, proxy, and context; and (4) moving toward person-centered care: empowerment and equity via an inpatient portal.

**Conclusions:**

An inpatient portal provides an opportunity to reconceptualize the medical record and how this information might be shared with patients while they are admitted to the hospital, such that they have more understanding as to what is happening in their care, which ultimately supports their well-being. The transition to a more transparent information-sharing culture in the Australian hospital context will take time. An inpatient portal is a critical step in facilitating this transition and creating more informational symmetry in the clinician-patient relationship.

## Introduction

### Background

Over the last decade, initiatives have been implemented across various countries to provide patients access to the medical record. Of these initiatives, Open Notes [1] is perhaps the most notable. Initiated in the United States in 2010, Open Notes is a call to action to provide patients access to their notes so that they can have more knowledge of, become more involved in, and have improved experience and outcomes in their health care. This initiative began as a pilot project to evaluate patient access to primary care notes [2]. As of 2021, it is now a US federal rule to provide patients access to their medical records [3]. Another key initiative is Planetree [4]. Founded in 1978, this international organization is dedicated to improving patient care from the patient perspective and has published numerous articles on the benefit of sharing the record with inpatients (refer to the study by Frampton et al [5]). Sweden’s Journalen is also another noteworthy initiative. Implemented in 2012, this system offers anyone from the age of 16 years access to their outpatient records from hospitals, primary care, mental health care, and dental care [6]. Alongside these initiatives, studies from the last decade have focused on and have been motivated by the incentive to share medical records with inpatients via portals for the reasons outlined earlier, that is, to enable the patient, as the patient-centered or person-centered discourse puts it, to become more “empowered” in their care [7-16]. These studies all underlined the inpatient portal’s beneficial impact on patient experience.

Studies (noted earlier) on the value and impact of sharing medical records with inpatients from both the patient and clinician perspectives are concentrated in the United States. These studies are generated from patient and clinician participant perspectives on hypothetical patient access to components of the medical record or from the actual clinician and patient experience of the sharing of and access to components of the record. These studies reported that clinicians are generally accepting of providing inpatients access to their records due to the benefits they provide patients. Also in these studies, clinician participants convey innovative ways the record could be shared with inpatients. However, clinician participants generally express caution that patient access to their records during their hospital stay may result in patient misunderstanding and anxiety as well as potentially compromise the quality of care. Patients generally report wanting access to their records and appreciate the potential transparency and inclusion in their care that this information sharing could provide. Significantly, studies involving the patient experience of the inpatient record report a decrease or no increase in patient anxiety [9,10,13,14] and therefore suggest a misunderstanding from the clinician perspective of the patient experience and a possible reduction of this experience to a limiting stereotype [17].

### Objectives of This Study

**Figure 1:**
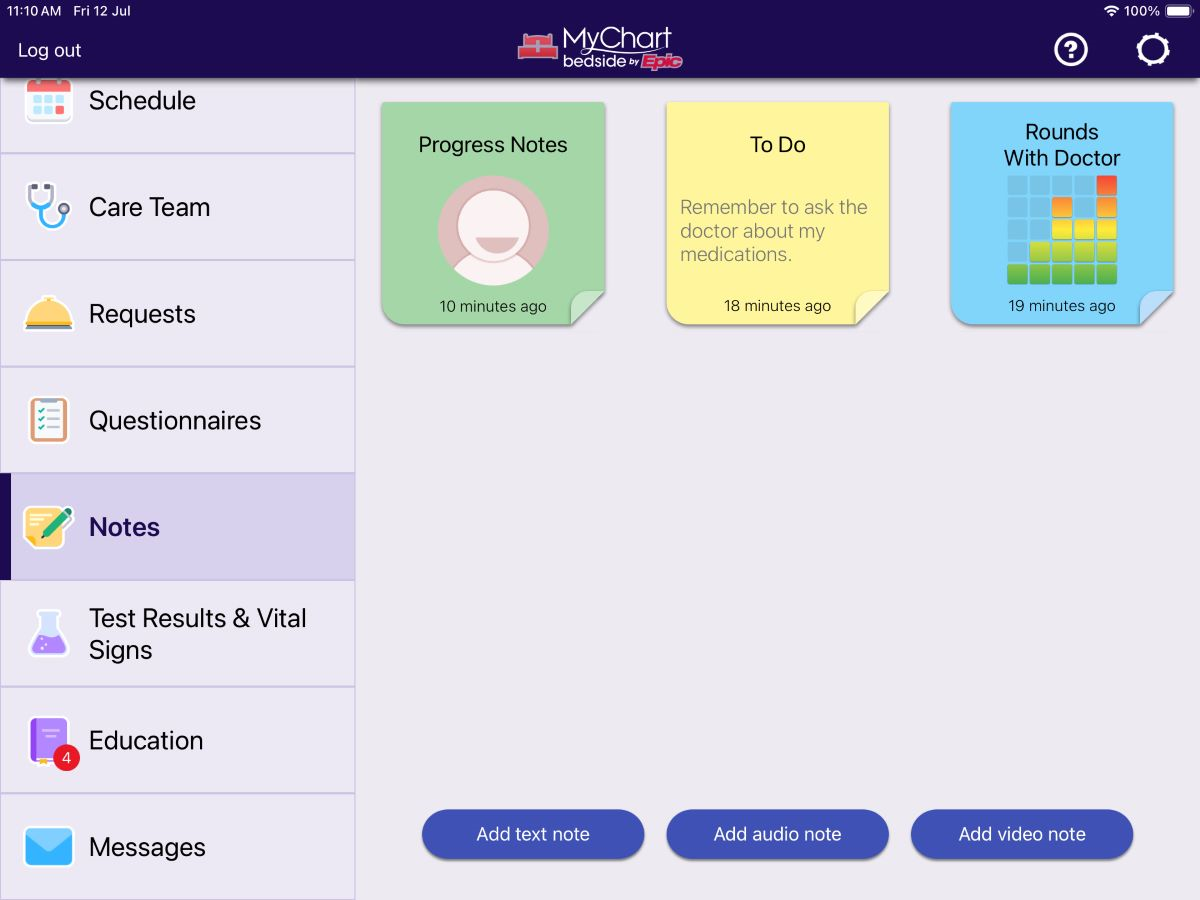
Screenshot of inpatient portal with menu bar, notes, and recording features as displayed on a tablet (MyChart is a registered trademark of Epic Systems Corporation).

**Figure 2:**
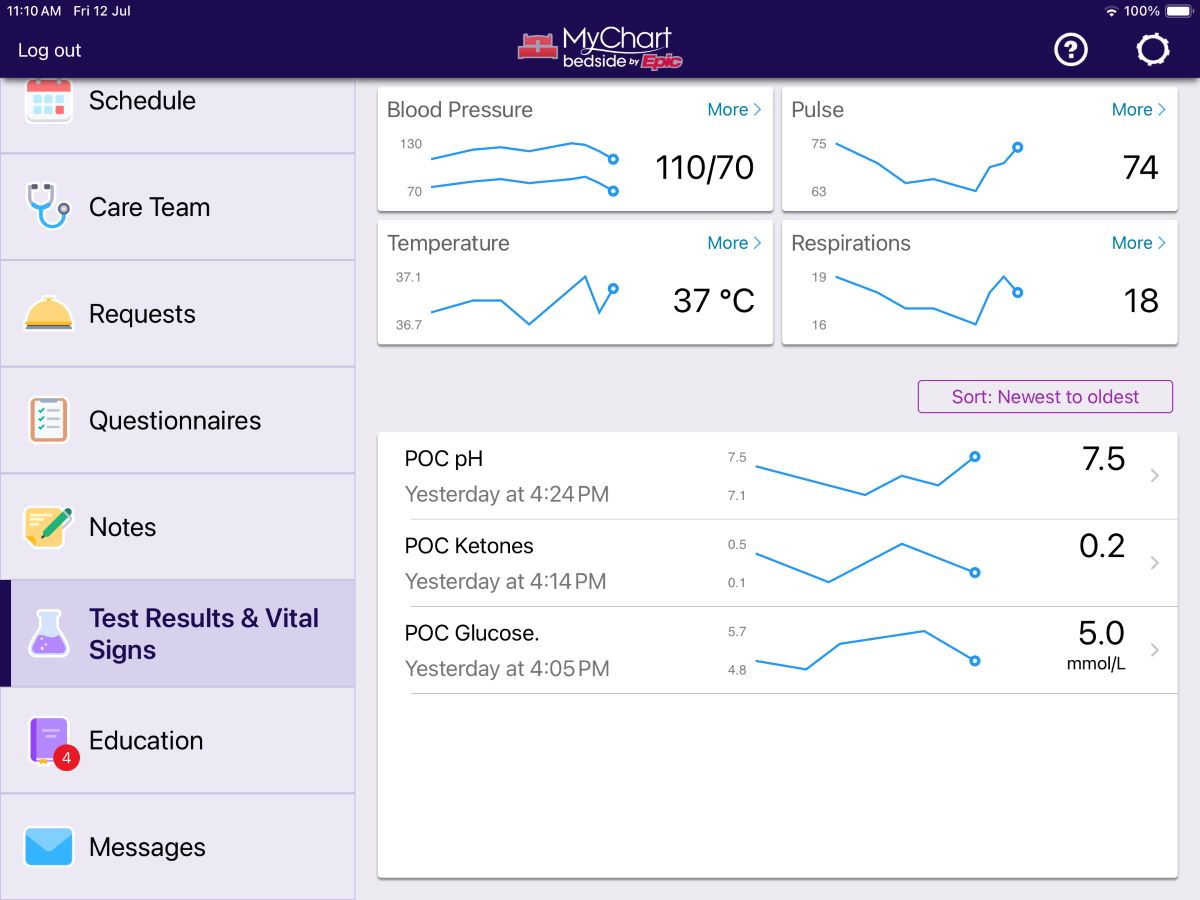
Screenshot of inpatient portal with menu bar and test result feature as displayed on a tablet (MyChart is a registered trademark of Epic Systems Corporation).

The motivation for our study was to explore patient, proxy (a nominated representative of the patient and in this study a partner involved in their care), and clinician perspectives of the MyChart Bedside inpatient portal within the Australian context before its future implementation. MyChart Bedside is an app by Epic Systems Corporation that extends the electronic medical record, providing patients access to aspects of their record (including notes, test results, and vital signs) while admitted to the hospital, via their smartphone or a hospital-provided tablet (Figures 1 and 2 show screenshots of the inpatient portal). To our knowledge, there are no studies of this kind exploring patient and clinician perspectives of patient access to their inpatient record in Australia.

In this qualitative study, we used the term *person-centered*, as person-centered discourse acknowledges the patient as more than solely a patient with a set of symptoms to be diagnosed and acted upon, and it acknowledges the carer as not only a carer but as a person with unique preferences and needs [18,19]. Initiatives that provide patients access to their records and studies focused on sharing information with inpatients (cited earlier) are aligned with the ethos of person-centered care. This ethos calls for health care to move away from the traditional paternalistic model of medicine characterized by an information asymmetry, where the clinician withholds information from the patient and proxy and positions them as a passive recipient in health care, to move toward a more balanced relationship characterized by greater transparency in information sharing between clinicians and patients and proxies, supporting greater patient and proxy collaboration with the clinician in their health care.

A significant contribution of this study to the studies in this area is that it approaches not only the patient and proxy but also the clinician through a person-centered lens. In this approach, the clinician is understood not solely in terms of their clinical function but also as a person who, just like the patient and the proxy, although for different reasons, may be vulnerable in health care. This study pointed to how recognition of clinician vulnerability may provide insights into their resistance to information sharing, which is the common result in studies exploring perspectives on patient access to their record. This study also shares with the person-based approach [20] the acknowledgment of the significance of the psychosocial dimension of its participants when exploring their perspectives. The person-based approach builds on user-centered and human-centered design and the principles of usability and acceptability and deploys a psychosocial lens to design and evaluate digital health interventions for behavior change and to enhance well-being. This study that has a different but related focus—an inpatient portal is also intended to enhance patient well-being—explored, where relevant, the psychosocial dimension of its participants when considering their perspectives and acknowledging their preferences about the acceptance of and vision of the sociotechnical system that the portal would generate.

This study was conducted within the context of the Royal Melbourne Hospital (RMH), a metropolitan, quaternary, and adult teaching hospital. Since August 2020, RMH has been offering its outpatients and their nominated proxies access to portions of their records, including select notes, test results, and medication lists via an outpatient portal (accessible via their smartphone or PC) referred to as “Health Hub.” The next logical step envisioned by RMH is to offer inpatients access to information pertaining to their hospital stay via an inpatient portal, MyChart Bedside. MyChart Bedside provides information affordances, including those that are the focus of this study—access to notes, test results, and the ability to message the care team and audio record consultations during the patient’s hospital stay. The primary incentive of our study was to determine the levels of receptivity and resistance among the clinician, patient, and proxy population to these affordances before the portal’s future implementation throughout the hospital. The primary research question that guided our study was “What is the foreseen value and impact of an inpatient portal from the patient, proxy, and clinician perspective?” Patients, proxies, and clinicians were interviewed to understand their perspectives on the portal’s functions, and this paper presents a reflexive thematic analysis of their perspectives. The exploration of patient, proxy, and clinician perspectives on the prospect of patients recording their consultations provides a novel contribution to the existing literature on patient portals that does not discuss this function.

This study builds on the knowledge generated in previous studies with particular attention to the psychosocial and cultural dimension of information sharing within the context of a hospital and the power dynamics involved between the patient and proxy and clinician. More generally, it points to why, despite the patient’s and proxy’s desire for more information on their health care and the clinician’s understanding of the value of sharing information with the patient and proxy, the transition to a more transparent, symmetrical relationship through information sharing between clinicians and patients and proxies is complex. This study describes this complexity while suggesting ways it can be addressed such that health care contexts, such as that of Australia, can move toward more person-centered care.

## Methods

### Recruitment

Clinician, patient, and proxy participants were recruited for interviews from areas within the hospital where the portal would later be implemented. These areas included ward 7B (hereafter referred to as the leukemia ward), hospital in the home (HITH) subacute, and HITH acute. These areas care for patients with blood cancers, notably leukemia, and extend inpatient care to a patient’s home for patients recovering from such conditions as having a stroke (HITH subacute) or breast cancer surgery (HITH acute). In total, 5 patients and 1 proxy were recruited from each of the HITH areas; 10 patients and 2 proxies were recruited from the leukemia ward. Recruitment of patients and proxies was facilitated by the nursing unit manager from the leukemia ward, a nurse educator from HITH acute, and a clinical coordinator from HITH subacute. Potential participants were selected via convenience sampling, that is, based on their mental and physical ability to participate in an interview and provide informed consent, their fluency in English (interviews were conducted in English as the hospital operates within an English-speaking environment), and their willingness to be interviewed. Author SS phoned the selected potential participants, and those interested in being interviewed nominated a date and time for the interview. More details on patient and proxy demographic characteristics (including context recruited from, age range, the highest level of education, employment status, comfort with technology, and understanding of their health condition) can be found in the Results section.

Nursing unit managers from the 3 areas and the chief medical information officer nominated the clinicians to be recruited. Their purposeful selection included a range of clinician types (including physicians, nurses, and allied health staff) and levels of seniority. A total of 9 (43%) of the 21 clinicians were recruited from the leukemia ward, 7 (33%) clinicians were recruited from HITH subacute, and 5 (24%) clinicians were recruited from HITH acute (refer to the Results section for clinician demographic characteristics, including context recruited from, clinician type, age range, level of education, and comfort with technology).

### Generation of the Dataset

#### Interviews

Participants were interviewed for 30 to 60 minutes via a videoconference platform. A semistructured interview guide was used. The interview explored the portal’s following functions: (1) access to the notes, (2) access to the test results, (3) ability to message clinicians, and (4) ability to record consultations.

Participants were invited to consider whether and how these information affordances were useful and how they would impact care. Interviews were recorded and transcribed.

#### Analysis

Reflexive thematic analysis was used in the analysis of the dataset. In this qualitative method, researchers acknowledge their epistemological position, that they coproduce knowledge with participants, and that knowledge is not “found” but generated [21]. This study took a contextualist epistemological position, which located knowledge as generated by people’s experiences and perspectives from a particular context at a particular point in time. Author SS took a largely inductive approach in the analysis of the dataset and conducted semantic coding of the transcripts in NVivo (Lumivero), describing the dataset and staying close to the participants’ voices. The codes and the subsequent themes were outputs generated from the dataset rather than preconceived through a theoretical framework. However, in the generation of a dataset, its analysis, and reporting, there is always a level of interpretation involved. The disciplinary background of author SS in the humanities and particular interest in ethics of sociotechnical systems informed the interpretation of the dataset. The disciplinary backgrounds of authors AB (nursing and informatics), BB (nursing), and TF (medicine and informatics) informed their review of the analysis and its finalization.

### Ethical Considerations

This study was approved as a quality assurance project by the RMH Office for Research in August 2022 (QA2022087) and required only verbal consent from participants. Participants received an information sheet detailing that their information would be deidentified in published material as well as in data storage. Patient and proxy participants were remunerated with gift vouchers.

## Results

### Participant Demographic Characteristics

The participant demographic characteristics are presented in Table 1 and Textbox 1. Patient and proxy participants were aged between 21 and 80 years, and clinician participants were aged between 21 and 60 years. Most of our patient and proxy participants had sound digital and health literacy: all patient and proxy participants reported a good understanding or a very good understanding of their health condition, and most reported being comfortable or very comfortable with technology. The education levels of the patient and proxy participants varied. Textbox 1 presents the clinician participant types recruited, grouped by each specific health care context. As can be seen, nurses were our most represented clinician participant type, followed by physicians.

**Table 1:** Demographic characteristics of participants.

Demographic characteristics			Patients and proxies (n=24), n (%)		Clinicians (n=21), n (%)
**Age range (y)**					
	21-30	2 (8)		6 (29)	
	31-40	6 (25)		7 (33)	
	41-50	3 (12)		6 (29)	
	51-60	6 (25)		2 (10)	
	61-70	6 (25)		—^a^	
	71-80	1 (4)		—	
**Highest level of education**					
	Secondary	9 (38)		—	
	Technical and further education	3 (13)		—	
	Bachelor	6 (25)		12 (57)	
	Postgraduate	6 (25)		9 (43)	
**Employment status**					
	Employed	16 (67)		—	
	Unemployed	4 (17)		—	
	Retired	4 (17)		—	
**Hospital context**					
	Leukemia ward	12 (50)		9 (43)	
	HITH^b^ acute	6 (25)		5 (24)	
	HITH subacute	6 (25)		7 (33)	
**Comfort with technology**					
	Not comfortable	2 (8)		—	
	Comfortable	12 (50)		10 (48)	
	Very comfortable	10 (42)		11 (52)	
**Understanding of health**					
	Good	16 (67)		—	
	Very good	8 (33)		—	

^a^Not available.

^b^HITH: hospital in the home.

**Types of clinicians recruited from each of the areas of future implementation.**

**Leukemia ward clinicians**

- Nursing unit manager
- Graduate nurse
- Assistant nursing unit manager
- Social worker
- Dietician
- Pharmacist
- Consultant
- 2 physicians

**Hospital in the home subacute clinicians**

- Clinical coordinator
- Home visit nurse
- Occupational therapist
- 2 speech therapists
- Physiotherapist
- Consultant

**Hospital in the home acute clinicians**

- Nurse educator
- 2 home visit nurses
- Pharmacist
- Consultant

### Thematic Overview

**Figure 3:**
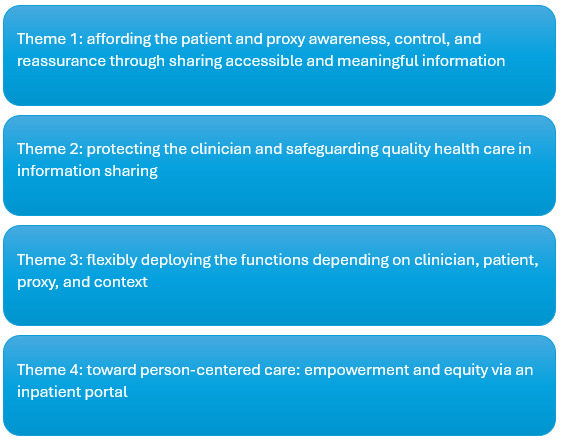
Thematic overview.

In total, 4 themes were generated from the dataset as conveyed in Figure 3: focused on the patient’s voice, theme 1 formed a major part of the analysis. It was concerned with how the inpatient portal’s functions can serve the patient and proxy, affording them awareness, control, and reassurance in their health care through accessible and meaningful information. After understanding what information the patient and proxy wants and the reason for it, we then need to understand how patient and proxy access to this information might impact the clinician’s practice. Theme 2 was focused on clinicians’ concerns about how the portal’s functions might impact them and their provision of care. Theme 3 considered how these functions can be flexibly deployed to serve the patient and proxy, protect the clinician, and safeguard quality care. Theme 4 presented an overall reflection on the value and impact of an inpatient portal from the patient, proxy, and clinician perspective, exploring the portal’s perceived strengths and limitations in relation to key concepts in person-centered care: *empowerment* and *equity*.

### Theme 1: Affording the Patient and Proxy Awareness, Control, and Reassurance Through Sharing Accessible and Meaningful Information

#### Overview

The most overwhelming factor generated in the patient and proxy interviews was that patients and proxies experience anxiety while admitted to the hospital due to the uncertainty about their situation. Patient and proxy participants explained that their anxiety is not so much a result of their health condition but their lack of awareness of what is happening in their health care. This theme was concerned with how functions that share meaningful information with the patient and proxy could bring them greater awareness of their situation and, by extension, a greater sense of control and peace of mind in their health care. Throughout this paper, *meaningful information* for the patient and proxy is understood as selective information regarding their health care that is tailored, relevant, and makes sense to them. Patients and clinicians conveyed the meaningfulness of information shared by the inpatient portal would depend upon the patient’s understanding of this information and, if necessary, whether it can be made meaningful by the clinician in conversation with the patient. Participants also considered the meaningfulness of this information could depend on added explicatory summaries; patient-friendly language; the selection of relevant information; and whether the patient wants this information generally speaking or at specific moments during their health care. Significant to this theme is the idea that the portal affords patients the ability to access information when they are ready because, as patient participants expressed, they experience periods of being unable to take information in due to the emotional and physical impact of their condition. This theme has been generated primarily from the patient and proxy perspective of functions that provide the option to share test results and notes and to record consultations. The clinician’s perspective on these functions contributes to this theme in a secondary capacity in terms of its degrees of alignment to or divergence from the patient and proxy perspective. The themes mentioned subsequently are labeled with the function they address and a patient or proxy quote or paraphrased perspective conveying a common patient and proxy perspective on the benefit of the function.

#### Theme 1.1 Test Results: It’s Good to Know Where You Sit Whether It’s Good or Bad

Patient participants from the leukemia ward considered it would be valuable if the portal could afford them more timely access to their results, rather than them having to wait for their clinician to share this information:

As a leukaemia patient, the one thing you’re always wanting to know is...your blood levels...those counts mean that...we’re stuck in hospital or we can...become day patients, which is what we all aim for.... First thing in the morning...pathology has already got your results, but you don’t know what they are.... If it’s on the app [portal...] you can...get the blood counts from...the night before.

Participant 38

Patient participants more generally explained that access to their results helps them understand their health, and, as many expressed, this understanding reassures them. One patient expressed, “It’s good to know where you sit; whether it’s good or bad.” Another stated that having access to these results would help them prepare for their consultations and feel more in control. Another explained that having access to their results could function as a kind of diary whereby they could track their health progress as they receive treatment. Accessibility was a key reason why patient participants were interested in having their results on the portal—having access to them whenever they needed them and being able to share this information with their care network (general physicians, family, and carers) was important to them. Some patient participants expressed the need for an explicatory summary or additional information to help them contextualize and understand their results:

It would be great to have...something that would explain...these results...It’s valuable in the sense that you can see things out of range, but you can’t put the context around it....You’re like, “I’m not quite sure what they’re looking for with that.”

Participant 43

Some clinician participants echoed the aforementioned perspective, stating that it would benefit patients to receive their results with an explanatory statement. A clinical coordinator stated the following:

I am on the health portal and love seeing my results, but I also know how to interpret them....If [they] came with explanations and general advice, I think that would be good.... Like what happens if my potassium is low? What do I do about it? My blood sugar is high. What do I do about it?

Participant 4

A more common clinician perspective was that results should not be shared with patients before being discussed with their clinicians. A pharmacist explained the following:

If you don’t have the skills to interpret it you shouldn’t be given information without explanation.

However, there were clinician participants who had no reservations regarding sharing results with patients and made no mention of the importance of the clinician first discussing these results with their patient or providing an explanatory statement. Echoing a patient perspective cited earlier, an assistant nursing unit manager from the leukemia ward stated the following:

A lot of them actually wake up at about three o’clock...in the morning because they know that that’s when results come back and they ask.... It would be good for them to just log on themselves and actually have a look, “Oh, I’m going to get some platelets today. Oh, my white cells are—I don’t think I can go home today,” because they know. They’re very well-informed. They know exactly what’s happening.

Participant 12

#### Theme 1.2 Notes: Knowing What Track You’re on Gives You Peace of Mind

Patient and proxy participants were interested in access to their progress notes for a greater understanding of and to keep track of their health care and to have something to refer to, particularly at times when it is difficult to take in or retain information from consultations:

You know that this is the track you’re on...you can...see if something’s changed.... If there was something major coming up and then I’d forgotten about it or missed something, I could go back into the notes and go, “Okay, no, we’ve decided that this is what we’re going to do”.... It’ll give you...peace of mind and definitely something to refer back to, because it can be so overwhelming. Sometimes you don’t retain all that information.

Participant 22

We would be able to look at the notes and if there was something that we just needed to reconfirm...or if...we didn’t quite understand what the doctor said.... I need to know exactly how she’s going, not only for her wellbeing but for mine...so I can do what I have to do as a carer looking after her.

Participant 11

A proxy participant, who is also a nurse and so understands what the progress notes involve, explained why access to their partner’s notes would have been helpful:

We couldn’t really visit him very much in hospital because visiting hours are restricted, so it would really help us to have access to check that everything was okay.... I guess it would just be extra reassurance.

Participant 1

However, some patient participants were aware that they would not be able to understand notes in their current form. A patient stated the following:

I was exposed to the hospital notes.... The language used is mainly for the medical department, so mainly it’s a jargon...a bunch of numbers and blood counts that...I couldn’t translate to tangible information for myself, so—yeah, it hasn’t been useful, but I’d like some more transparency there.

Participant 22

Some patient participants suggested a patient-friendly, summary record alternative to the clinician-facing notes, as they assumed the latter would be difficult to understand and therefore not useful.

Clinician participants were comfortable sharing admission and discharge notes with patients and proxies, but most were reluctant to share progress notes. Those open to sharing progress notes echoed the patient suggestion of a patient-facing note that could function as a summary record, filtering out aspects of the notes that could offend, confuse, or distress the patient. Some clinician participants considered how certain sections of the notes, such as those from the ward rounds and assessment notes, could be of value to the patient. A physician stated the following:

If I say, “...the next couple of days, the plan is to do a CT...to give you some transfusions—” this and that...I don’t think much of that would sink in.... I almost...want to give them a...a list to...say, “Okay, this is what’s happening today. This is what’s happening tomorrow.”...That’s what’s captured in a ward round note.... If they have access to the ward round note, it’ll be nice for them to see what’s on the agenda...to understand what’s happening for their illness....

Participant 26

A speech therapist stated the following:

If I did a swallowing assessment...and had a report, I would write it in a patient-friendly manner and...share it, whereas if it was just a progress note with no home therapy program...then I would maybe just keep that for the other clinicians.

Participant 17

There was a minority (2/21, 10%) of clinician participants who were open to sharing notes and wrote them as if a patient could access them. A physiotherapist explained that this was how they were trained to write notes. A social worker explained that they write their notes as if a patient could read them but simultaneously expressed discomfort at the prospect of a patient reading their notes.

#### Theme 1.3 Recording Consultations: I’d Feel More Informed and Relaxed About My Care Because I Wouldn’t Need to Remember Everything

Most patient and proxy participants were interested in recording their consultations due to similar reasons for wanting access to their notes—the view that it is difficult to take information in when you are unwell and that having this record of information would bring awareness, control, and reassurance in their health care. A patient stated the following:

I’d feel more informed...and more relaxed about my care, because I wouldn’t need to remember everything. I’ve felt anxious some of the time because I figured I was being told something very important, but especially when I’d just come out of ICU...I could barely relate what had happened to me in terms of what the doctor had said.... I didn’t feel confident...talking to the doctor. I needed someone else...there to take in what they were saying.

Participant 39

This perspective conveys how the recording function could be beneficial for patients who do not have someone with them who can help them process information communicated to them. Another common reason for patient interest in recording consultations was to be able to share this information with their care network. Patient participants expressed that sometimes when they try to relay consultation information to their family, they are not always able to do so accurately, if at all. The following quote is from the proxy, who is also a nurse:

Because [he...] was unable to speak...he was unable to tell me what happened in those consultations.... That was really hard. He had a whole team of people come and see him, but he was unable to...relay that information. So it would be great to have them recorded.

Participant 1

One patient participant stated that they would not need to record their consultations because they have “pretty good recall.” A proxy participant explained that they would not want this because they trust their partner’s clinicians. Formerly a police officer, this participant associated recording with surveillance.

Despite having reservations, many clinician participants could see the value in providing patients with the option to record their consultations. They considered it could function as a record for patients to return to or share with their care network if they were not proficient in English. They also considered it could be valuable as a form of education and echoing the patient participants, a record for when patients cannot take their consultations in, for when their recall is limited, and to share with families absent from consultations. A graduate nurse stated the following:

When the doctors come in...it can be quick.... They don’t process the information a hundred percent....To have that...conversation recorded that they can go back and listen to...might be helpful because sometimes you need to hear information a few times to process it.

Participant 3

There was a minority (2/21, 10%) of clinician participants who, due to their ethos of transparency, had no problem with patients recording their consultations. There were others—also a minority (3/21, 14%)—who were opposed to it, due to the vulnerability they felt in relation to it. Most (16/21, 76%) clinician participants had “mixed feelings” regarding this prospect.

### Theme 2: Protecting the Clinician and Safeguarding Quality Health Care in Information Sharing

#### Overview

It was immediately apparent in the interviews that just as patients and proxies experience anxiety due to lack of awareness and therefore control in their health care, clinicians experience vulnerability in sharing information with patients. This vulnerability could be a result of the clinician’s level of seniority, expertise, training, experience, work culture, the medicolegal implications of their field, or a combination of all these factors. This theme focuses on the clinicians’ concern regarding patients recording consultations and accessing progress notes. Clinician participants considered how patient access to these information affordances might result in scrutiny of their practice, which could negatively impact them as well as compromise the quality of care provided. This theme is developed solely from the clinician’s perspective. The following subthemes are labeled with the function they address and a common clinician perspective.

#### Theme 2.1 Recording Consultations: This Would Make a Lot of Clinicians Uncomfortable

Clinician participants recognized that enabling patients to record their consultations would benefit patients but felt uneasy about this. They explained that they did not want their words taken out of context to be misinterpreted, misappropriated, and vulnerable to any medicolegal implications. A speech therapist considered that if consultations were recorded, they (the therapist) might overwhelm the patient with information to safeguard themselves rather than giving the patient the information that is most meaningful for them at that moment in their health care. They stated the following:

We should be very aware of what we’re saying, and...comfortable enough with what we’re saying that we’re happy...to be recorded....Some of the things that I probably said when I was working three or four years ago are no longer actually accurate because of new evidence so I think there would need to be some safeguards....What can happen with those recordings, where can they go? Is it just accessible through the app or is it going to be uploaded by the patient somewhere else?

Participant 31

A physician considered the following:

It’ll be helpful to play back those recordings and try and understand...things a bit better...and ask questions. So, I can see that that’s pretty valuable. It does make me...nervous when patients say, “Can I record things?”...That is something that would make a lot of clinicians uncomfortable.... Perhaps, that is more of a reflection of us, and what we’re worried about with our practice than what is best for the patient.... A lot of concern...would be the medico-legal implications of recorded information, and information, perhaps, being taken out of context....

Participant 26

Several clinician participants stated that they would support the recording of consultations if there was consent from the clinician and regulations to ensure information would not be misinterpreted and misappropriated. There was also the view that this function should only be deployed in certain situations where it was needed.

#### Theme 2.2 Sharing Progress Notes: I’d be Worried About the Self-Filtering That Might Happen and What Impact That Might Have on Clinician Communication and Patient Care

In their consideration of sharing progress notes, a speech therapist reflected as follows:

It’s hard because it’s like a culture in the hospital, you wouldn’t expect for the notes to be shared, so it’s a whole change of thinking.

Participant 17

This perspective that sharing notes with patients would require a cultural shift and an evolution of thinking perhaps explains why many clinician participants were resistant to the idea of sharing progress notes. Importantly, several clinician participants understood why patients may want access to the notes and stated they would want access if they were patients; however, they also felt reservations about sharing these notes with patients when considering the clinician’s perspective.

Clinician participants were generally concerned about how patient and proxy access to progress notes might negatively impact clinical practice and patients and proxies. Many clinician participants explained that these notes are a mode of communication between clinicians and include differential diagnoses, queries, social issues, and observations on mood and behavior that may confuse, offend, or distress the patient or proxy. They explained that if they were to share these notes, they would need to change the way they write, and this may impede communication with their colleagues and degrade the quality of care. A nurse explained the following:

I’d be worried about people not potentially fully documenting things [...because they are] worried what the patient might see or how they might react to what they’ve read.... That’s certainly an area that’s going to take...more exploration as the program unfolds.

Participant 2

A dietician expressed the following:

I’d just be worried at the level of self-filtering that might in fact happen and what impact that might actually have on communication between teams and...patient care....There’s perhaps a question about the purpose of why they might need access to everything when it comes to their notes....

Participant 40

As with the clinicians’ perspective on recording consultations, there was the idea that it should be optional to share specific notes depending upon the patient, the clinician, and the situation, rather than a default sharing of all notes.

### Theme 3: Flexibly Deploying the Functions Depending on Clinician, Patient, Proxy, and Context

#### Overview

Throughout the interviews, particularly when interviewing clinicians, it became clear that for the functions to be of value to and to have a beneficial impact on the clinician, patient and proxy, and health care context, they would need to be flexibly used. The flexible use of functions meant that they would not be used in the same way in every situation, but their use would depend upon the clinician, patient, proxy, and context. Rather than a one-size-fits-all approach, it was conveyed by clinicians that certain notes and results (rather than others) may be beneficial to share, and in some cases, recording consultations would be appropriate. The flexible use of functions would enable the clinician to adapt the information-sharing system according to their shifting levels of comfort, the shifting needs of the patient, and the particularities of the context.

This theme introduces a new function—the messaging function—and draws from both patient and clinician perspectives. The interviews conveyed that if the messaging function were to be implemented, it would need to be done flexibly, depending on the clinician, patient, and context. Furthermore, regulations would need to be put in place for its purpose and to set patient expectations regarding clinician response. Many clinician participants, particularly physicians, were opposed to this function due to the extra work it would entail in responding to messages. Patient participants also expressed concern that this function would place too much burden on physicians. However, there were clinicians who could see how messaging could be an effective means of communication with patients in their respective roles as clinical coordinators, speech therapists, and occupational therapists from the HITH subacute context. There was unanimous agreement among patient and nurse participants from the leukemia ward that messaging would be an effective means of patient-nurse communication and would support quality care. The following subtheme is labeled with the function it addresses and a key patient perspective from the leukemia ward that was echoed by nurse participants from this same health care context.

#### Theme 3.1 Messaging: It Would Be So Good for Nurses and for Us as Patients Because Nurses Could Go to Where They Need to Be

Nurse participants stated after a patient buzzes them, they must go into their room to determine what they need, whereas messaging would immediately convey to them what patients need, and they would be able to determine who to attend to first depending upon the urgency of the situation. They stated messaging would be particularly useful in situations where they would otherwise need to put on protective wear to go into the patient’s room, which takes time. One nurse explained the following:

On our busy days, our buzzers are just going off the hook [which...] adds...stress to the nursing staff. Having something like that to [...know] the things that are urgent and the things that aren’t...would help.

Participant 12

Another nurse stated the following:

If you could get a message on your rover [nurses’ communication device] telling you exactly what they need...it would save time....

Participant 3

Patient participants shared the nurses’ perspectives, adding the insight that messaging would be beneficial if they needed to let their nurse know that they were in a critical condition:

My temperature can be raging at 41 and I can be close to death, and I push the button, or I need a bottle of cordial, and I push the button. And the nurse...doesn’t know whether I’m nearly dead or whether I want the bottle of cordial....I think the app would be brilliant.... It would be so good for the nurse, and so good for us as patients because then the nurses could go to where they really need to be....

Participant 41

The interviews made clear how messaging could improve patient-nurse communication in the leukemia ward, enabling nurses to more effectively manage their patients’ needs and potentially reduce nurse and patient stress.

### Theme 4: Toward Person-Centered Care: Empowerment and Equity Via an Inpatient Portal

#### Overview

This theme explores the perspectives of the patient, proxy, and clinician participants on the value and impact of an inpatient portal through 2 critical and related concepts in person-centered care: *empowerment* and *equity*. The adage *knowledge is power* was voiced throughout the interviews when considering the potential value of the portal’s information affordances. The terms *empowerment* and *control* were often expressed—and were often expressed together—by participants when they considered the portal’s value. A significant insight generated by the interviews was that if a person shifts from the position of the clinician to that of patient or proxy, they become disempowered in their access health care information. Within the discourse on person-centered care in digital health, as concerns the context of this study, the proposition is that an inpatient portal could facilitate greater equity for the patient and proxy in their relationship with their clinician and in their health care through providing patients and proxies with accessible and meaningful information. This theme explores this proposition and highlights the potential strengths and limitations of the portal concerning the concepts of empowerment and equity. The following subthemes are partially labeled with participant quotes.

#### Theme 4.1 The Portal Empowers Patients to Be More in Control Like an Equal Person in Their Care

Reflecting on the value of the portal, a nurse stated the following:

That’s the thing that stood out to me the most...it would be really good for them.... It empowers them to be more in control and like an equal person in their care.

The term *empowerment* became especially poignant when clinicians considered when they had been patients and had experienced an immediate disempowerment in their role reversal. A physician explained the following:

When they change roles...from a healthcare professional to a patient, even though...they’ve done so many years of study and training, immediately...being a patient, just kind of disempowers them in a way that’s very confronting....As the treating team, you have all the information...you just feel like, “Yep, we know what’s going on.” But as a patient, you are relying on someone to tell you what the results are and where you’re at with your treatment.... To just feel like you’re in control...that’s what the app [portal] might help with.

Participant 26

This perspective conveys the dramatic shift in access to information and, by extension, power that occurs when a clinician becomes a patient. The clinician knows what’s happening (to a degree), and the patient depends on the clinician for this information. As the nurse who was the proxy participant in this study expressed, she would have wanted access to her husband’s progress notes and the ability to record his consultations, so she could have a better understanding of what was happening to him. This would have reassured her. This participant nurse did not hesitate in expressing her desire for this information, despite also being a clinician and therefore familiar with the Australian hospital information-sharing culture, which restricts patient access to such information. As the aforementioned quote conveys, perhaps the reason why there was no reservation in accessing this information, as is normally the case from the clinician’s perspective, was because, in the position of patient or proxy, one is disempowered. Reflecting upon the value of the inpatient portal, a patient participant stated the following:

You’d just feel a little bit in control. At the time, we’re totally out of control.

Participant 9

Another explained the following:

I think it’s empowering to know more about your condition. If you know more, you understand more, you can make better decisions.... When you know more, you’re just more in control.

Participant 27

#### Theme 4.2 The Portal Highlights and Marginalizes the Less Equitable Population

Considering the value of the portal, the proxy participant, who is also a nurse, reflected on caring for her husband, who is a physician and had just had a stroke:

I’m very aware that for us, as awful as it’s been, it’s been fairly seamless because we understand what’s going on. I can’t imagine how terrifying it must be for people who don’t understand.... If people are...informed and they feel like they can ask questions, I think it [the portal] could only be a good thing. Knowledge is power and people feel better equipped to support their loved ones if they’ve got all the information because it’s very intimidating, this whole talk about MRIs and CTs, and clot retrieval.... We have to embrace the technology.... It’s easy for English speakers and people who don’t have a pre-existing disability.... It’s not that easy for everyone.

Participant 1

This perspective raises the issue of equity of access for people who are not proficient in English or have a disability that impedes their intake of information. The fact that the portal would only be accessible in English concerned some clinician participants. Clinician, patient, and proxy participants were also concerned that the portal would not benefit older adults or those who were not technology proficient. A consultant considered the following:

Is it empowering? Yeah, if it gives the information in an accurate way.... But...there’s still that bias towards the tech-savvy.... Older [people from] non-English speaking backgrounds [...this] population who would most benefit—one, would not be able to use it, and two, the materials we provide are not in their language of choice. So [it...] highlights and actually marginalizes the less equitable population.

Participant 10

This perspective shared by several clinician and patient participants points to the limitations of the portal’s affordances.

## Discussion

### Overview

This study’s patient and proxy participants wanted health care information accessible via an inpatient portal to increase their control and, by extension, decrease their anxiety during their hospital stay. Clinician participants generally wished to support inpatient access to information via the portal but also expressed resistance and uncertainty in this area. Clinicians will need more experience in information sharing via inpatient portals in health care contexts like that of Australia to understand how it might impact care. A flexible deployment of the inpatient portal’s functions could support exploration in this area. Clinician and patient participants shared ideas on how to reconceptualize the record such that it is accessible and meaningful to the patient and proxy. This study highlighted how an inpatient portal can help address the information asymmetry and power imbalance that characterizes the patient-clinician relationship.

All patients and proxies interviewed were highly interested in access to notes, and test results via an inpatient portal. All but one participant was interested the ability to record their consultations. Patient participants explained that, when unwell, it is difficult to process and retain information and having the option of accessing meaningful information—information that is relevant and makes sense to them—regarding their health care when they are ready to process it would be valuable. When considering information sharing via the inpatient portal, 3 key clinician perspectives were generated. The first 2 perspectives were held by a minority (5/21, 24%) of clinician participants. They involved either (1) an openness without reservation to sharing information with patients and proxies or (2) a complete resistance to sharing information with patients and proxies. The third perspective, held by most (16/21, 76%) clinician participants, involved an openness to information sharing with patients with reservations as to what, how, between whom, and in what context information should be shared. This perspective generated theme 3 focused on the flexible deployment of the functions depending upon the clinician, patient, proxy, and health care context. The importance of flexible deployment of the portal’s information affordances, particularly in relation to high-risk situations, is noted elsewhere [22]. Furthermore, as another study has stated, when sharing health information, there should not be a one-size-fits-all approach, as different people need different information at different times and in different contexts [7].

This study contributes to existing studies on sharing medical records via an inpatient portal by acknowledging that how this sociotechnical system is configured will be determined not only by the individual patient’s need for awareness, control, and reassurance in their health care but also by the clinician’s need to protect their practice. Some clinician participants self-reflexively considered their attitude toward sharing inpatient notes or enabling the patient to record consultations. These participants, when reflecting on their attitudes toward information sharing, considered that these attitudes could be a result of their education, their work culture, and the broader medicolegal implications of their field. The significance here is that just as patient and proxy anxiety is a critical component to consider in the case of the value of information sharing through an inpatient portal, clinician vulnerability and indeed, anxiety, in relation to this process must also be acknowledged. As 1 clinician participant conveyed sharing inpatient notes is not part of the existing culture of the hospital and requires a new way of thinking, and as another clinician participant expressed, it will need exploration.

Shifting hospital culture to accommodate new attitudes and exploring new approaches to information sharing with inpatients will take time. Indeed, in the American context, OpenNotes charts the evolution of information-sharing culture in health care over half a century [1]. In the Australian context, where the prospect of sharing the record with inpatients is still in its infancy, a flexible sociotechnical system is needed while clinicians adjust to the changing information-sharing culture and become more comfortable with becoming increasingly transparent with patients and proxies. However, clinicians will need a high level of awareness when making the decision not to share information with patients and proxies. They will need to know if this decision is motivated by a limited attitude toward the patient that disempowers them and excludes them from their health care, or whether this decision is motivated by concern for the patient’s well-being or because of the complexity of the treatment that requires time before meaningful information can be conveyed to the patient [17,22]. It has been noted that policies are needed to guide clinicians in the sharing of sensitive information in this transforming information-sharing culture [12].

The evolution of health care information sharing will require clinicians to continually reflect upon their attitudes. As raised in the Introduction section, a common clinician perspective is that if inpatients have access to their notes and tests, they will become anxious. However, as noted previously in this paper, studies exploring the inpatient experience of such information have shown that patient anxiety does not increase and indeed, in some cases, decreases [9,12,14]. This common clinician misunderstanding that access to information will increase patient anxiety has been argued to be the result of reducing the patient to a stereotype, which in turn produces a *hermeneutical injustice*: where a person’s right to understand their situation is impeded [17,23]. What is overwhelmingly reported in this study is that patient anxiety is the result of not knowing what is happening more so than knowing what is happening. As 1 patient participant so clearly stated, “It’s good to know where you sit; whether it’s good or bad.”

This study has generated significant insights into how clinicians are inquiring into their current practice and reflecting upon why they may be resistant to certain forms of information sharing, despite understanding the value it would have for the patient and proxy. As a physician reflecting on clinician resistance to recording considered, “Perhaps, that is more of a reflection of us, and what we’re worried about with our practice than what is best for the patient.” This reflection highlights the tension between professional expectations and wanting to provide patients with greater access to information. This clinician self-reflexivity is a critical component in the movement toward greater information symmetry and in addressing the power imbalance in the patient- and proxy-clinician relationship. As insights from clinician participants conveyed, the impetus for developing a more symmetrical relationship is most clear when the clinician places themselves in the position of the patient or proxy and understands the disempowerment the patient and proxy experiences due to limited access to their health care information. Indeed, compelling insights came from clinicians who drew from their direct experience of the reversal of roles and their recognition that the patient and proxy is disempowered in their relation to the clinician.

A perspective was raised in this study that the portal would highlight and marginalize less equitable populations, including older people and people who do not speak English. However, a study conducted in the United States has noted that less equitable populations benefit from patient portals the most: Black people, people who have low education levels, and those who do not speak English at home reported access to the record as more important than White people, people with high levels of education and native English speakers [24]. However, this idea is contradicted in a more recent study in the inpatient context that reported that African American patients used the inpatient portal less than White patients [25]. For our study, many of our patient and proxy participants had only a secondary school education, which indicates that a desire for health care information is not dependent on being highly educated. It is noteworthy that a patient who has difficulty speaking English or has low digital literacy may share their portal’s information affordances with their care network, such as their family. This idea of the value of a digital platform being accessible by multiple parties involved in a patient’s care is emphasized elsewhere [26].

A key insight generated from this study was that patients and proxies want access to information that is *meaningful*—information that is tailored, relevant, and makes sense to them. This perspective led to participants’ conceptualization of how the record could be shared. Clinician participants considered what aspects of the notes would be useful to the patient, such as the ward round notes and assessments. Patient and clinician participants considered how the notes, if they were shared, would need to be in patient-friendly language and a summary form. This idea of the summary note has been raised in another study that reported that information sharing enabled by patient portals may call for a redesign of notes [7]. This idea of reconceptualizing the record has been highlighted in another study, which states the importance of clinicians “writing notes that patients will read” [22]. A patient participant from an earlier study exploring patient experiences on access to their records explained, “I read until I found technical stuff, and then I would jump over it...The parts I read, and I understood, were interesting and beneficial” [13]. This perspective is echoed by patient perspectives generated in this study that conveyed that unless patients understand the information, the information will not be useful. Likewise, clinician participants considered the importance of sharing useful information with patients; 1 clinician participant stated that information is not necessarily a good thing and could result in information overload. As noted in a previous study, selecting information that is meaningful for the patient and that does not overwhelm them will require an exploration of how best to present this information to the patient via the portal’s interface [12].

Another significant contribution of this study to the literature on inpatient portals is the exploration of clinician and patient perspectives on enabling patients to record consultations. Although these perspectives have been explored in studies focused solely on the topic of recording [27], the option to record consultations has not been explored as a function of the inpatient portal. Clinician participants considered how it would be valuable for the patient to record consultations if they were not proficient in English so they could return to the consultation or share it with their care network. Clinician and patient participants considered the recording function useful to capture the details of an intervention or education, to share a consultation with the patient’s care network, and to give the patient time to process the information because when unwell it is difficult to take information in, particularly when one has, as several patient participants put it, “chemo brain.” Most clinician participants expressed resistance to enabling this function, cautioning that before such a function could be implemented, there should be safeguards in place to protect the clinician from any medicolegal consequences that could result from such a practice. Research in health care recording in the Australian context details patient rights and legal responsibilities [28]. In Victoria, if the consultation is in person, the patient has a legal right to record it and does not require the consent of the health care provider; consent is advised to protect the patient-clinician relationship. However, for the patient to share the recording with others, clinician consent is legally required. To record and share telehealth consultations in Victoria, the patient is legally required to have the consent of the clinician [28]. Neither patient nor clinician participants in this study were aware of this legal framework. Perhaps if they were, the prospect of recording consultations would be normalized, rather than being a subject of desire from the patient’s perspective and a subject of fear from the clinician’s perspective.

This study enabled patient, proxy, and clinician participants to reflect on how they want information shared in the inpatient context and the type of person-centered sociotechnical system they want to create to support the well-being of everyone involved in the health care context but most importantly the patient. Within the Australian context, the transition to a more transparent patient-proxy-clinician information-sharing relationship is still very much in process. An inpatient portal is a valuable step in this process.

### Principal Results

The participants’ consideration of an inpatient portal involved a reconceptualization of the medical record and information-sharing practice within the hospital. Participants considered how the portal’s potential influence on and redesign of information sharing could result in enhanced patient-clinician communication and more transparency in the provision of care, which could contribute to patient and proxy well-being. This study highlighted how the clinician-patient information asymmetry correlates with the power imbalance involved in the clinician-patient relationship. Alongside the influence of an inpatient portal that initiates a rethinking of the medical record and how it can be shared with the inpatient, clinician self-reflexivity is a valuable step in addressing this power imbalance and information asymmetry. This study also underlined that clinician’s vulnerability needs to be acknowledged in the evolving information-sharing culture and that the portal’s functions could be flexibly deployed with the intention to protect the clinician’s practice. Finally, this study reported the value of recording consultations from the patient and proxy perspective and the messaging function from the patient and nurse perspective within particular contexts.

### Limitations

Study participants were recruited from only 3 contexts within the hospital; participants from other contexts may have provided different perspectives. Only patients and proxies fluent in English were included in the sample due to the need to conduct the interviews in English. Furthermore, all patient and proxy participants had a good understanding of their health, and most were comfortable using technology. This could be because the types of patients and proxies who agree to an interview focused on health care technology are usually people who have reasonable health and technology literacy. The study sample could therefore reflect how the less equitable patient and proxy population is excluded from the health care context.

### Comparison With Prior Work

This study echoed results reported in previous studies: patients desire the information affordances of an inpatient portal to support their well-being, and clinicians understand the value of these information affordances but express caution regarding certain information sharing. This study has contributed to the knowledge of patient and clinician perspectives on inpatient portals through its exploration of the power dynamic that characterizes the patient-clinician relationship as well as drawing attention to clinician vulnerability in information sharing with patients. It considered how an inpatient portal might assist in transforming the hospital’s information-sharing culture to support person-centered care. It provided a novel contribution to the literature on inpatient portals by exploring perspectives on the recording function. Finally, it explored perspectives on an inpatient portal from within the Australian context, pointing to how, in this context, health care is still in the process of transitioning to person-centered care.

### Conclusions and Future Work

Participants explored how information could be shared via an inpatient portal. Patient and proxy participants expressed their desire for the portal’s information affordances to support their well-being. Clinician participants reflected on their resistance and receptivity to information sharing with patients and proxies. Clinician participants expressed that there needs to be further exploration of information sharing via an inpatient portal to understand its value and impact. This study is the first stage of a 2-part research project; our next step is to explore the experiences of patients using the inpatient portal in a recent implementation in the leukemia ward. It is important to note that this recent implementation of the inpatient portal in the leukemia ward has not been informed by the results of this study but was realized as an independent project, and this implementation does not include access to progress notes nor the recording or messaging functions. However, once the 2 stages of this study are published, their combined results will inform future implementations of the inpatient portal throughout the hospital and potentially lead to patient access to more comprehensive information affordances within the inpatient context.

## Abbreviations

HITH: hospital in the home
RMH: Royal Melbourne Hospital

## Data Availability

The datasets generated during and analyzed during this study are not publicly available to protect participant privacy.
